# Mathematical Models for Immunology: Current State of the Art and Future Research Directions

**DOI:** 10.1007/s11538-016-0214-9

**Published:** 2016-10-06

**Authors:** Raluca Eftimie, Joseph J. Gillard, Doreen A. Cantrell

**Affiliations:** 1 Division of Mathematics, School of Science and Engineering, University of Dundee, Dundee, UK; 2 CBR Division, Defence Science and Technology Laboratory, Porton Down, Salisbury, UK; 3 Division of Cell Signalling and Immunology, School of Life Sciences, University of Dundee, Dundee, UK

**Keywords:** Mathematical immunology, Advances since 2006 and future trends, Innate and adaptive immunity, Multiscale interactions, 92-02, 92B05, 92C50

## Abstract

The advances in genetics and biochemistry that have taken place over the last 10 years led to significant advances in experimental and clinical immunology. In turn, this has led to the development of new mathematical models to investigate qualitatively and quantitatively various open questions in immunology. In this study we present a review of some research areas in mathematical immunology that evolved over the last 10 years. To this end, we take a step-by-step approach in discussing a range of models derived to study the dynamics of both the innate and immune responses at the molecular, cellular and tissue scales. To emphasise the use of mathematics in modelling in this area, we also review some of the mathematical tools used to investigate these models. Finally, we discuss some future trends in both experimental immunology and mathematical immunology for the upcoming years.

## Introduction

**Fig. 1:**
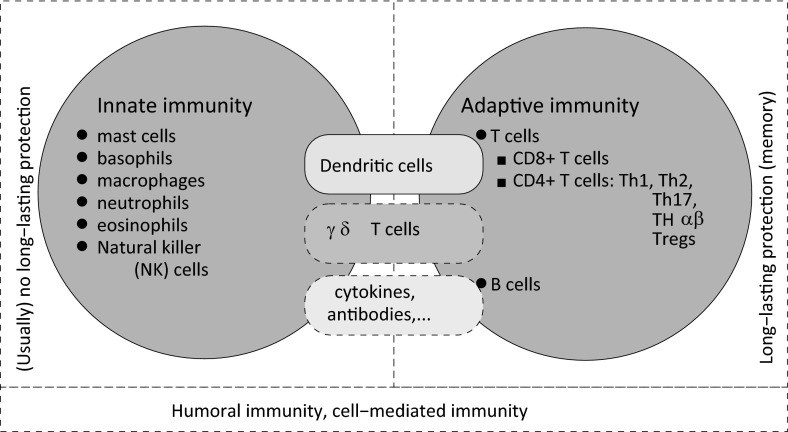
Brief description of various components of the innate and adaptive immune responses. Both the innate and adaptive immunity include humoral aspects (e.g., antibodies) and cell-mediated aspects (e.g., cytokines)

The immune system is subdivided into two main subsystems, the innate system and the adaptive system, which are connected via the action of various cells (e.g., dendritic cells), cytokines, antibodies, etc.; see Fig. 1. These two subsystems generally cooperate to ensure the protection of the host (Meraviglia et al. 2011). The innate immune system focuses on the physical and chemical barriers formed of cells and molecules that recognise foreign pathogens. The adaptive immune system focuses on the lymphocytes’ action to clear these pathogens. The innate immune dendritic cells (DCs), which connect the two immune subsystems, recognise pathogen molecules via invariant cell-surface receptors and then display their antigens on their surface to be recognised by the T cells of the adaptive immune response (Murphy 2012). In addition to the DCs, the two subsystems can be also connected via the action of a particular type of T cell, called the $\gamma \delta$ T cells, which are considered both a component of adaptive immunity (since they develop memory) and of innate immunity (since some of their alternative T cell receptors may be used as pattern recognition receptors) (Meraviglia et al. 2011). We remark here that the notion of immune memory has been associated for a long time with only the adaptive immune response (as mediated by the lymphocytes). However, very recent experimental results have shown also the existence of a type of innate immune memory associated with macrophages (Yoshida et al. 2015) or with NK cells (Borghesi and Milcarek 2007). Another distinction between the innate and adaptive immunity is related to specificity: the innate immune response is considered to be non-specific (relying on a large family of pattern recognition receptors), while the adaptive immune response is considered to be very specific (relying on clonally distributed receptors for antigens, which allow cells to distinguish between, and respond to, a large variety of antigens). Finally, both the innate and adaptive immunity include humoral components (e.g., antibodies, complement proteins and antimicrobial peptides) and cell-mediated components (that involve the activation of phagocytes and the release of various cytokines); see Fig. 1.

Many of the complex interactions between the innate and adaptive immune systems and the pathogens that trigger the immune responses (interactions which occur via complex networks of cytokines and chemokines) have started to be revealed in the last 10–15 years, especially because of the advances in genetics, high-throughput methods, biochemistry and bioinformatics. A 2011 review in *Nature Reviews Immunology* (Medzhitov et al. 2011) highlighted some of the fundamental advances in immunology since 2001: e.g., improved understanding of Toll-like receptor signalling, improved understanding of immune regulation by regulatory T cells, improved understanding of myeloid-derived suppressor cells. In particular, one of the most cited immunology papers over the last 10 years is a review of monocyte and macrophages heterogeneity by Gordon and Taylor (2005). Other significant advances made in the last 10 years were in the areas of cancer immunology and immunotherapy (Chen and Mellman 2013; Kalos and June 2013), inflammation (Kim and Luster 2015), autoimmunity (Farh et al. 2014), infection (Rouse and Sehrawat 2010; Romani 2011), and metabolism (Mathis and Shoelson 2011; Finlay and Cantrell 2011).

These recent advances in immunology have led to the development of a large number of mathematical models designed to address some of the open questions unravelled by these advances. Particular interest was given to mathematical models for the activation of T cells, models for the molecular pathways involved in the activation, migration and death of various immune cells (e.g., T cells, B cells, neutrophils), models for cancer–immune interactions, as well as models for the immune response against various infectious diseases such as HIV, malaria, tuberculosis, etc. Over the last 10 years, some of these mathematical models have been summarised and reviewed in various contexts: choosing the correct mathematical models for describing an immune process (Andrew et al. 2007), reviewing models for T cell receptor signalling (Coombs et al. 2011), models for various intracellular signalling networks (Janes and Lauffenburger 2013; Cheong et al. 2008; Kholodenko 2006), the evolution of mathematical models for immunology (Louzoun 2007), non-spatial models of cancer–immune interactions (Eftimie et al. 2010a), agent-based models of host–pathogen interactions (Bauer et al. 2009), multiscale models in immunology (Kirschner et al. 2007; Germain et al. 2011; Cappuccio et al. 2015; Belfiore et al. 2014). This large number of reviews of various types of mathematical models, published in both immunology and mathematical journals, is a testimony of the great interest and fast advances in this research field.

**Fig. 2:**
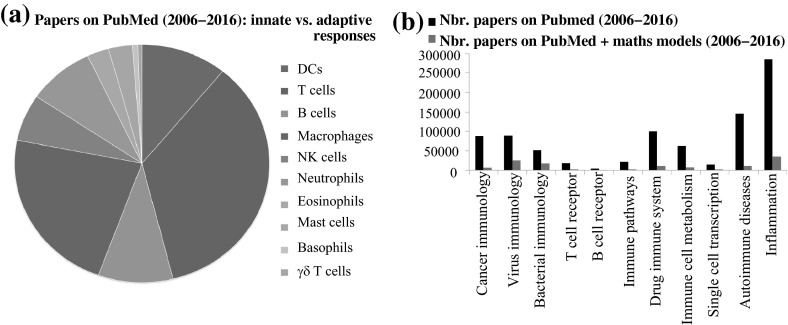
**a** Pie-chart description of the number of papers published on PubMed between 2006 and 2016, which focus on different types of cells belonging to the innate and adaptive immunity. **b** Number of papers published between 2006 and 2016 on PubMed, which deal with various aspects of the immune response: from cancer immunology, to viral and bacterial immunology, immune pathways, etc. The data used to create these figures were obtained from the PubMed database, using the words that appear on the figures labels as the search words. For the *red bars* (*grey on black*/*white * prints) shown in **b**, we also added “mathematical model” to the search words. Note that the mostly experimental studies described by the *black bars* and the theoretical/mathematical studies described by the *red bars* follow similar patterns: a larger number of studies on inflammation and on virus and bacterial immunology, and a much lower number of studies on T cell and B cells receptors, or on single cell transcription (Color figure online)

In this study, we aim to give a review of mathematical immunology over the past 10 years (i.e., since 2006). To this end, we will cover the breadth of progress rather than any particular research area in great detail. Nevertheless, given the spread of this field, we will only offer a brief description of some of the mathematical models. To ensure minimal overlap with previous reviews published since 2006, we will focus on the most recent models, the techniques developed to investigate these models, and the potential impact of the mathematical results to designing new experimental studies. Since a brief PubMed search showed that a relatively equal number of papers have been published in the last 10 years on either innate or adaptive immune cells (see Fig. 2a), we decided to include in our review mathematical models for both innate and adaptive immune responses. In addition, since the immunological research over the past decade covered a variety of immune responses associated with basic immune activation (via T cell and B cell receptors), viral and bacterial infections, immune response to cancers, inflammation, autoimmunity, etc. (see Fig. 2b; and our previous discussion on recent advances in immunology), we will review mathematical and computational models that were derived to address questions regarding these various immune aspects. Moreover, we will discuss future trends in mathematical immunology, as well as emphasise areas where mathematical immunology methods may be applied beyond their original context.

*Role of mathematical models in immunology* There are many viewpoints in regard to the purpose of developing mathematical models to describe immunological phenomena: from explaining existing observations and generating new hypotheses that can be tested empirically (Ankomah and Levin 2014), to understanding which assumptions in the model are useful and generate outcomes consistent with data [and thus help discriminate between different immune hypotheses (Antia et al. 2005) to uncover basic mechanisms driving some phenomenon (Shou et al. 2015)], organising data resulting from experiments (Shou et al. 2015), offering a selection criteria for ideas that could be tested experimentally in vivo or in vitro (thus reducing the cost and the time associated with performing large numbers of experiments) (Seiden and Celada 1992), evaluating the feasibility of an intuitive argument (Shou et al. 2015), or making theoretical contributions to the knowledge related to immunological systems [by demonstrating the possibility of some outcomes as a results of specific interactions in a particular type of environment, and by suggesting further theoretical problems (Caswell 1988)]. Caswell (1988) distinguished two general purposes for mathematical models: to offer some general theoretical understanding for a theoretical problem (and this understanding does not need to depend on model validation), and to help make predictions (which depends on model validation).

*Model validation* Throughout this review, whenever we refer to “model validation” we actually mean [as discussed in Oreskes et al. (1994)] that models are partially confirmed by showing agreement between observation and prediction [complete confirmation of biological models being impossible (Oreskes et al. 1994)].

As stated in Rykiel (1996), the belief that complete model validation is impossible is based on the idea that model falsification should be critical for science. However, Karl Popper’s falsifiability criterion (Popper 1965) (i.e., a theory is scientific only if it makes predictions that can be falsified), which has been already challenged by other philosophers and scientists (Thagard 1988; Mentis 1988; Rykiel 1996), cannot be easily applied to the subtleties of modelling biological phenomena, where many unobservable quantities (e.g., interaction rates) cannot be easily quantified, thus leading to models that cannot be rejected directly (Rykiel 1996) (at least not with our current knowledge). Moreover, as emphasised by Caswell (1988), experimentalists recognise that no experiment represents the last word on the subject, and that an experiment can be usually understood in the context of other experiments that manipulate different factors (and thus might contradict the original empirical experiments), making it difficult to validate mathematical and computational models in immunology.

*Parameter estimation* In mathematical and computational immunology, many researchers use parameters published in the literature to justify the results of their simulations (both parameters measured experimentally, and parameters taken from other published mathematical and computational models). However, this represents a major issue, since very few laboratories measure and estimate kinetic parameters; see, for example, the studies in Boer and Perelson (2013), Gadhamsetty et al. (2015), and their discussion on the difficulty of interpreting kinetic data. Moreover, even in this case, the parameters are estimated for specific experimental systems/models and might differ from study to study (depending on the estimation method used, and on the characteristics of the experimental model, e.g., the inbreed strain of the laboratory mouse used in experiments, or the cell line used in experiments) (Boer and Perelson 2013; Laydon et al. 2015). The only rigorous approach (very expensive and time consuming), which could lead to results that could have predictive power, is to estimate in a laboratory all parameters required by a mathematical/computational model (describing a specific system). For simplicity, throughout the next four sections, whenever we refer to models for which parameters were obtained from the literature (in contrast to parameters calculated experimentally) we actually mean that those parameters were not estimated in a rigorous manner and thus they might not depict accurately the kinetics of the system. The studies where kinetic parameters were measured in a laboratory will be emphasised separately throughout this review [see, for example, Sect. 5, where we discuss the computational and theoretical approaches in Zheng et al. (2008) and Henrickson et al. (2008)].

**Fig. 3:**
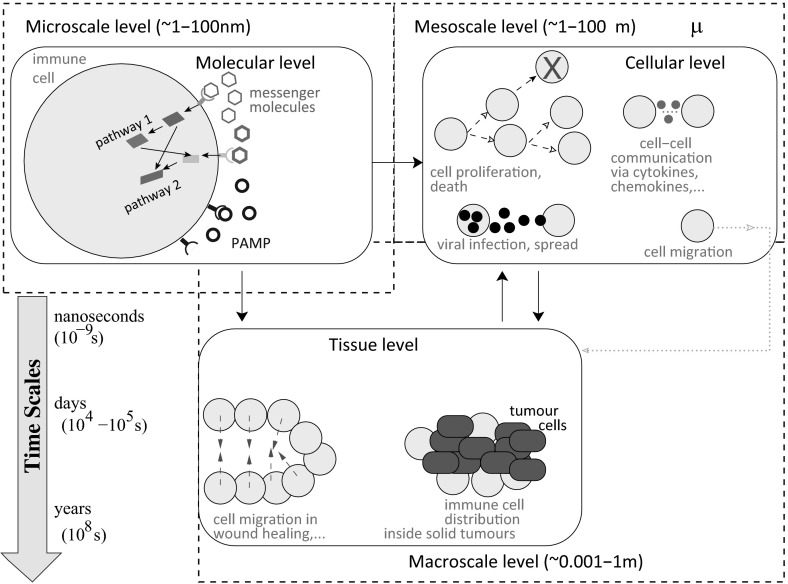
(Colour figure online) Caricature description of examples of immune processes at molecular, cellular and tissue levels. A different classification of multiscale immune processes focuses on the spatial ranges at which these processes take place: microscale, mesoscale and macroscale. Note the overlap between cellular- and tissue-level processes with the mesoscale spatial level. This is the result of migration of cells between different tissues (e.g., from the lymphoid tissue where cells get activated to the peripheral tissue where pathogens reside). Immunological processes also vary across temporal scales: from nanoseconds (for some molecular processes) to days and even years (for some cellular and tissue-level processes)

**Fig. 4:**
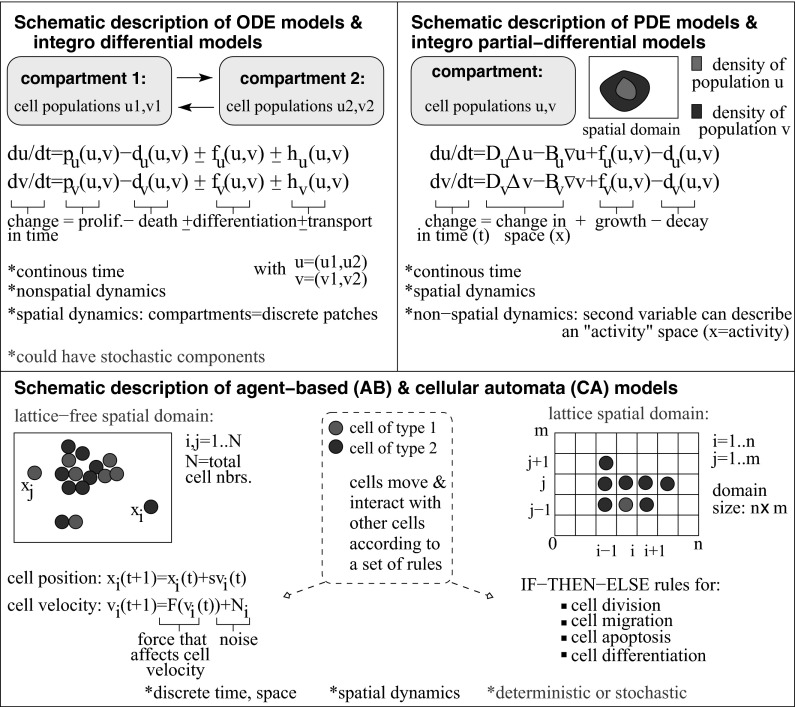
(Colour figure online) Schematic description of various types of mathematical models derived to investigate immune dynamics (see also “Appendix 1”). In many cases, these types of models are combined; for example CA models can be coupled with PDE models (which are discretised), PDE models can be coupled with ODE models, CA models can be combined with AB models, etc. There are also many other types of models not depicted here; e.g., probability models (e.g., quantifying the probability of encounters between T cells and dendritic cells (Celli et al. 2012)), algebraic models describing the binding and unbinding of B cell receptors (Fellizi and Comoglio 2015). All these models are usually coupled with ODEs, to describe multiscale immunological phenomena. For a review of various modelling frameworks in immunology see Kim et al. (2009)

*Multiscale aspects of mathematical models in immunology* To capture the complex multiscale dynamics of the immune responses, the review will cover both innate and adaptive immunity across the molecular/genetic scale, cellular scale, and tissue/organ scale (see also Fig. 3). We emphasise that in addition to these spatial scales, immunological processes also span a range of temporal scales: from nanoseconds for peptide binding, to seconds/minutes for the production and degradation of cytokines involved in immune cells communication, and to days and months for the proliferation and death of some long-lived immune cells (e.g., memory T cells). However, throughout this study we will neglect the temporal scale (since many of the mathematical models neglect it), and we will focus mainly on the spatial scale. At each of the spatial scales, we will review some mathematical models derived to address some of the questions that have dominated the immune research over the past 10 years. For example, at the molecular scale, the past years have seen the immunology research being focused on: (i) understanding the mechanisms for Tcell receptor binding to peptide major histocompatibility complex (MHC) molecules and B cell receptor binding to antigens, and (ii) understanding the different signalling pathways involved in the activation and functionality of immune cells. This translated into a wide range of mathematical models that have been developed to investigate these aspects in the context of adaptive immune cells [both (i) and (ii)] and innate immune cells [mainly (ii)]. At the cellular scale, the mathematical models followed the advances in the immunology research of diseases (both viral and bacterial), autoimmunity and cancer. The review will summarise models that investigate (i) only the role of innate immune cells, (ii) only the role of adaptive immune cells, and (iii) models that combine both innate and adaptive immunity. At the tissue scale, the few mathematical models for the immune response focused mainly on the immunological aspects of wound healing and scaring, as well as on the immune cells distribution inside solid tumours or granuloma. Finally, we discuss multiscale models, which investigate immune processes that take place across various spatial scales. The variety of mathematical models derived to capture all these different immunological processes is depicted in Fig. 4 (with the models briefly described and compared in “Appendix 1”). For completeness and accessibility, we also added a glossary of mathematical terms in “Appendix 2”.

We start each subsection by presenting a list of references emphasising the variety of studies published after 2006 on that particular topic. Then, we discuss in more detail two arbitrarily chosen studies: one study which emphasises the power or limitations of experimentally validated models and one study which offers a theoretical understanding of a model derived to simulate immunological phenomena.

Note that while we review only research published after 2006, we will also refer to a few types of general papers published before 2006: (i) older papers that put forward or emphasised general ideas regarding the importance and multiple roles of mathematical models in biology; (ii) older papers on the philosophy of science, which we refer to when discussing our take on model validation; (iii) older experimental papers that put forward an important immune concept that we need to refer to (especially in the context of evolution of experimental research).

The article is structured as follows. In Sect. 2 we review mathematical models that address questions regarding the molecular-level immune interactions. In Sect. 3 we review mathematical models for cellular-level immune interactions. In Sect. 4 we review mathematical models for tissue-level immune interactions. In Sect. 5 we give an overview of some of the models derived to investigate immunological phenomena that takes place between different scales. We conclude with Sect. 6, where we discuss the applicability of these models to a broader immunological context, and possible future trends.

## Models for the Molecular-Level Immune Dynamics

Two areas in molecular immunology where progress has been made in the past 10 years (see also Fig. 2b), and which generated the development of various mathematical models, are: (i) the mechanisms for T cell receptor (TCR) binding to peptide MHC molecules and B cell receptor (BCR) binding to antigens; (ii) the different signalling pathways involved in cell functionality. Note that while models (i) are developed in the context of adaptive immunity, models (ii) are developed mainly for innate immunity. In the following, we will briefly review the types of mathematical models derived to address these immune aspects.

(i) *Models for TCR and BCR binding and diversity* The central aspect in the generation of an adaptive immune response is the binding of TCRs to peptide major histocompatibility complex (pMHC) molecules (Coombs et al. 2011) and the binding of BCRs to antigens, which leads to the activation of T cells and B cells. Aberrant regulation of T cell and B cell activation not only impacts the fight against infections, but it can also lead to autoimmunity (Chakraborty and Das 2010). One class of models derived to describe the biochemical signalling that follows the TCR/pMHC binding are the kinetic proofreading models, which were introduced to explain how T cells can discriminate between ligands based on the dissociation time of ligand–receptor interactions and were recently reviewed in detail by Coombs et al. (2011). Over last 10 years, these models have been used, for example, to calculate rigorously parameter values from experimental 2D and 3D data (Qi et al. 2006), to investigate the sensitivity of TCR to self and agonist ligands (by combining the concepts of kinetic proofreading, cooperative interactions between self and agonist ligands that amplify signalling, and feedback regulation of Lck kinase) (Wylie et al. 2007), to investigate the role of CD4 and CD8 co-receptor molecules on TCR signalling (Artyomov et al. 2010), to investigate the timescale associated with T cell responses based on stochastic versus deterministic (i.e., equilibrium) assumptions (Currie et al. 2012), to investigate the bistability dynamics caused by positive and negative feedbacks in TCR signalling (Lipniacki et al. 2008), or have been generalised to incorporate spatial movement of TCR and pHMC particles along the cell membrane (Burroughs et al. 2006), or to investigate the spatial segregation at the immune synapses of small proteins (e.g., TCR/pMHC) and large proteins (e.g., CD45) on the surface of T cells and NK cells (Burroughs et al. 2011). In fact, all these mathematical and computational models and many more other models not mentioned here focus in some way or another on quantifying the TCR:pHMC dissociation rates for different antigens, or the maximal T cell response obtained at saturating pMHC concentrations, with the goal of understanding and predicting T cell and B cell activation during infection or cancer immune therapies (Gannon et al. 2015; Dushek et al. 2014; Nag et al. 2010; Lever et al. 2014).
  Another aspect investigated recently with the help of mathematical models is the estimation of the size and diversity of T cell receptors (TCRs) and B cell receptors (BCRs) (Stirk et al. 2008; Johnson et al. 2012; Baltcheva et al. 2012; Lythe et al. 2016), or the use of T cell receptor excision circles (TREC) to quantify thymic output (Dool and Boer 2006).
  Next, we will discuss in more detail two models: one which emphasises the importance of modelling to discriminate between different assumptions following comparison with experimental data and one theoretical model which investigates the role of stochastic fluctuations in TCR signalling.
Baltcheva et al. (2012) developed two non-spatial mathematical models (of ODE type) and used them to understand the kinetics of the concentration of DNA molecules obtained via the AmpliCot technique, which measures the diversity of DNA samples through quantification of rehybridisation speed of polymerase chain reaction (PCR) products. The two models in Baltcheva et al. (2012) (a simple second-order kinetics for the formation of homoduplexes of complementary strands of DNA, and a more complex heteroduplex model for these DNA strands) were then used to fit the variable for the proportion of fluorescent material in the sample to available experimental data. Confidence intervals for the parameter values were also computed, using bootstrap replicates of the data. The results showed a better fit for the heteroduplex model, which can capture the nonlinearity in the data. It should be noted here that the simple second-order kinetics model had only one parameter, while the more complex heteroduplex model had 5 parameters. However, the authors used the likelihood ratio test for nested models to show that the improved data fit observed with the heteroduplex model was significant for all three data sets used in model validation (Baltcheva et al. 2012). These types of results emphasise the usefulness of mathematical models on allowing us to discriminate between models/assumptions capable to fit or not the data [aspect considered important by Oreskes et al. (1994)].
Lipniacki et al. (2008) derived a stochastic kinetic proofreading model that includes competition between positive and negative feedbacks for T cell receptors (on a single cell). To investigate the possibility of bistable behaviour, the authors also derived a deterministic limit of this stochastic model. The deterministic model does exhibit bistable behaviour as the number of activating peptides is varied, which is the result of negative and positive feedbacks. Moreover, computational results showed that in the bistable case the deterministic model cannot approximate adequately the averaged stochastic trajectories. On the other hand, in the monostable case, the deterministic trajectories approximate the stochastic averaged trajectories. Overall, these theoretical results emphasise the qualitatively different dynamics exhibited by similar deterministic and stochastic models.
  The majority of mathematical models for T cell and B cell activation are either described by deterministic ODEs or are stochastic computation models (e.g., Monte–Carlo simulations, where reaction probabilities approximate the kinetics of network components). Overall, pairing mathematical and computational modelling with experimental results has ensured a better understanding of T cell signalling (Chakraborty and Das 2010). Nevertheless, not all results of these models were consistent with published data (see the discussion in Lever et al. (2014) in regard to models for TCR-MHC binding). One possible reason for this inconsistency between analytical and experimental results is incomplete available data (Lever et al. 2014). The spatial aspects of the local cell membrane environment, which seem to play an important role in TCR function (Burroughs and Merwe 2007), make it even more difficult to obtain adequate data. Moreover, the models that seem to explain the data are the phenotypic models that incorporate a minimal set of assumptions, and not the mechanistic models based on a large number of assumptions (Lever et al. 2014; Francois et al. 2013).
(ii) *Models for cell signalling pathways* The response of cells to external signals is encoded by the spatial and temporal dynamics of the signalling pathways activated by membrane receptors (Kholodenko 2006). Dysregulation of these pathways leads to diseases that range from developmental diseases, to cancer, diabetes, etc. (Kholodenko 2006). Over the last 10 years various mathematical models have been developed to investigate some of these pathways in the context of the innate immune responses (Cheong et al. 2008; Vodovotz et al. 2008), or in the context of the adaptive immune responses (Perley et al. 2014). Since most of the B cell and T cell receptors discussed previously can initiate intracellular signalling by the activation of protein tyrosine kinases (Murphy 2012), some of these models also investigate signalling through T cell receptors (Perley et al. 2014). In regard to innate immunity, one of the most investigated signalling pathway is the NF-$\kappa$B, which controls the regulation of genes involved in immune and inflammatory responses (Bonizzi and Karin 2004). There are actually two such pathways: a classical activated pathway mostly involved in innate immunity and an alternative activated pathway involved in adaptive immunity (Bonizzi and Karin 2004). The classical pathway (which is activated in monocytes, macrophages and other innate cells by specific pathogen-associated molecular patterns) is triggered by ligand binding to tumour necrosis factor type 1/2 receptors (TNFR1/2), T cell receptors, B cell receptors or the Toll-like receptors and leads to a persistence of inflammatory responses and promotion of cell survival (Nishikori 2005). The alternative pathway is activated by B cell activating factor belonging to the TNF family, Toll-like receptors or CD40 ligand and seems to be important in development and maintenance of secondary lymphoid organs (Bonizzi and Karin 2004). The majority of mathematical models developed to investigate the NF-$\kappa$B pathway focused only on the classical pathway (Cheong et al. 2008; Basak et al. 2012; Williams et al. 2014; Yilmaz et al. 2014; Tay et al. 2010). These models studied different aspects of the pathway: from a minimal model of 3 coupled ODEs derived to understand oscillations in the nuclear-cytoplasmic translocation of the NF-$\kappa$B transcription factor (Krishna et al. 2006), to more complex models derived to understand the feedback between components of the pathways such as I$\kappa$B$\alpha$, I$\kappa$B$\beta$ and I$\kappa$B$\varepsilon$ with the help of 24 ODEs describing the time evolution of molecular species of this pathway and one PDE for the diffusion of TNF-$\alpha$ molecules (Cheong et al. 2006). This later study showed that NF-$\kappa$B is sensitive to a wide range of TNF-$\alpha$ concentrations. The model in Cheong et al. (2006) was later generalised in Tay et al. (2010) to include stochastic effects, and comparison with high throughput quantitative data revealed that not all cells responded to TNF-$\alpha$. Other ODE models have been derived to exemplify parameter fitting methods and sensitivity analysis for parameters describing the rates cell signalling pathways (Fujarewicz et al. 2007), or to exemplify the use of bifurcation theory to obtain a better understanding of the system’s response to TNF-$\alpha$ (Wang et al. 2012). In addition to these ODE models, there are other models that investigate the dynamics of the molecules, receptors and genes in the NF-$\kappa$B pathway using an agent-based approach (Pogson et al. 2006). For a recent review of these NF-$\kappa$B models, see Williams et al. (2014).
  It should be mentioned that there are many more mathematical models that focus on other signalling pathways. For example, a few models were derived to help understanding lipopolysaccharide (LPS) signalling via Toll-like receptor 4 (TLR4) in macrophages during inflammation and sepsis (Rivière et al. 2009; An 2008). Some of these models were described by ODEs (Rivière et al. 2009), while other models considered an agent-based approach (An 2008). For a more detailed review of models for signalling pathways activated during inflammation, see Vodovotz et al. (2008). Also in the context of innate immunity we mention the existence of models for signalling pathways activated following infections with different pathogens [e.g., *Francisella tularensis* (Leander et al. 2012)], models for signalling pathways (e.g., PI3K) that control migration and polarisation of neutrophils (Onsum and Rao 2007), models that try to elucidate the pathways involved in the crosstalk between various cytokines that regulate immune responses, such as IFN-$\gamma$ and IL-6 (Qi et al. 2013), models for gene regulatory networks that control genetic switching between cell fates, such as the GATA genes in hematopoietic stem cells (Tian and Smith-Miles 2014), or models for the regulation of signalling pathways in innate immune cells following viral infections (Tan et al. 2012) and the optimal control of the innate response (Tan and Zou 2015).
  However, not all models in the literature focus on intracellular signalling pathways in the context of healthy immune cells. For example, there are a range of ODE models that investigate various pathways involved in metabolism and disease: from glutathione metabolism (Reed et al. 2008), to folate-mediated one-carbon metabolism (Reed et al. 2006), arsenic metabolism (Lawley et al. 2014), or glucose metabolism (Chew et al. 2009). For a review of these metabolic models see (Nijhout et al. 2015). We emphasise that the recent focus of experimental research on the metabolic regulation of the immune response (Ganeshan and Chawla 2014) will see the adaptation of these mathematical models to the reality of metabolic pathways inside immune cells.

The majority of models discussed in the previous paragraphs are described by relatively low numbers of equations. However, there are models that try to incorporate all components of the signalling networks, thus being described by hundreds and even thousands of equations (Danos et al. 2007). These complex models are investigated numerically with the help of software such as BioNetGen, COPASI, Kappa or NFsim—the last one generalising an agent-based kinetic Monte–Carlo method (Faeder et al. 2009; Sekar and Faeder 2012; Danos et al. 2007; Sneddon et al. 2011; Tóth et al. 2015; Hoops et al. 2006).

Next, we will discuss in more detail one such complex model, which can offer mainly a theoretical understanding of the system. In addition, we also present a (slightly simpler) model which was validated against some experimental data and further used to make predictions (in the absence of experimental understanding) regarding the synergy between the two components of a signalling pathway and its effect on immune response to infection.

- An example of a complex ODE model investigated with the help of BioNetGen was introduced in Barua et al. (2012) to describe the signalling pathways activated by the binding of BRC to antigens. The rule-based model, which incorporated six signalling proteins (BCR, Lyn, Fyn, Csk, PAG1 and Syk) and was described by 1122 equations, was investigated using bifurcation analysis to show bistability dynamics of the Lyn tyrosine kinase involved in early BCR signalling events. The bifurcation parameter was the strength of antigen signal. To ensure that the bifurcation persists when varying the 25 parameters considered essential for model dynamics, the authors also performed a sensitivity analysis. The results of the model seemed consistent with known effects of Lyn and Fyn deletion on BCR signalling: Lyn deletion caused a delayed and enhanced activation of Syk, while Fyn deletion caused impaired Syk activation (Barua et al. 2012). It should be noted that, in general, very large models are not easily investigated in terms of bifurcation analysis. Moreover, we emphasise that despite that it has become easier to model very large signal transduction networks, we are still not close to a mechanistic understanding of the effects of various components of the networks on the final outcome. To this end, it is necessary to look for reduced models within the larger network (e.g., network motifs) and to try to understand these reduced models first (Prasad 2012).
- Despite evidence (provided by various experimental data on the CR3/TLR2 crosstalk during *F. tularensis* infection) confirming that Lyn kinase and PI3K are essential components of the CR3 pathway that influence TLR2, detailed information about the components responsible for upstream signalling is not available experimentally. To test some hypotheses regarding the components of the CR3 pathway responsible for the inhibition of ERK activity, Leander et al. (2012) developed a mathematical model of Complement Receptor 3 (CR3) and Toll-like Receptor 2 (TLR2) signalling in response to the intracellular pathogen *F. tularensis*. The authors first showed that the mathematical model (described by ODEs) was consistent with experimental results in Dai et al. (2011) for the concentrations of various signalling molecules. With the help of numerical simulations, the authors predicted that the Akt and Ras-GAP components of the pathway played an important role in ERK inhibition. Uncertainty and sensitivity analysis (via Latin hypercube sampling) was performed to assess how uncertainty in model parameters (the majority of which being taken from the published literature) affected model consistency with experimental data. The authors concluded that the model was consistent with experimental data over a wide range of parameter values (Leander et al. 2012). This analysis also allowed the authors to identify those molecules (incorporated into the model equations) which seemed to be important for the CR3-mediated inhibition of the ERK component of the pathway: the over-expression of TLR2 or Ras, or reduced expression of Ras-GAP.

Finally, we remark that the majority of mathematical models studying molecular-level processes are described by non-spatial ODEs. While the use of these equations renders the investigated problem more tractable and the model more easy to investigate, it may not capture all biological phenomena. For example, Chaplain et al. (2015) showed that, while a 2-equation ODE model of the Hes1 transcription factor cannot exhibit the experimentally observed oscillations in both mRNA and protein concentration levels, a spatially-explicit PDE version of the model can account for these oscillations (via the Hopf bifurcation it exhibits). Therefore, more research is necessary to discern between the types of mathematical models that can be applied to model specific biological phenomena.

## Models for Cellular-Scale Immune Dynamics

To overview the mathematical models derived to describe cell-level immune dynamics, we will present separately some models that investigate (i$^{\prime }$) only innate immune responses, (ii$^{\prime }$) only adaptive immune responses, and (iii$^{\prime }$) immune responses involving Dendritic Cells (DCs), which act as a bridge between the innate and adaptive immunity. We will also briefly summarise some models that investigate the interplay between innate and adaptive immune responses (without the explicit incorporation of antigen-presenting cells). For this cellular-scale dynamics, in addition to the models describing direct cell–cell interactions, we will also focus on models describing the interactions between cells and cytokines, antigens and viruses (since, despite the molecular action of antigens/cytokines/viruses, the majority of mathematical models treat them as object similar to cells, where the interactions are averaged). We emphasise here that in contrast to the models discussed in Sect. 2, the models for cellular-scale dynamics are described by fewer equations. This allows for a more detailed mathematical investigation of the models, as will be discussed at the end of this section.

(i$$^{\prime }$$)
*Models for cell-level dynamics during innate response* The dynamics of the innate immune response has been investigated with the help of mathematical models, for example, in the context of bacterial infections alone (Malka et al. 2010; Smith et al. 2011; Mochan et al. 2014; Zaitseva et al. 2014; Gillard et al. 2014; Day et al. 2011), viral infections alone (Saenz et al. 2010; Canini and Carrat 2011), viral and bacterial infections (Smith et al. 2013), chronic wound inflammation (Nagaraja et al. 2014) or more general inflammation (Dunster et al. 2014), and immune responses to cancer (Webb et al. 2007; Knútsdóttir et al. 2014). These mathematical models range from simple deterministic ODEs (Day et al. 2011; Canini and Carrat 2011) and PDEs (Knútsdóttir et al. 2014; Webb et al. 2007), to stochastic models (Gillard et al. 2014).
  Some of these mathematical models have been validated quantitatively and qualitatively against available data and then investigated numerically. Other studies on the innate immune response combined numerical and analytical tools to obtain a deeper understanding of the nonlinear dynamics of the models. Next, we will discuss in more detail two such complementary approaches to model and investigate innate immune responses.

- Mochan et al. (2014) introduced an ODE model that described the interplay between the populations of *S. pneumoniae* in the lungs and blood, the concentration of phagocytes (neutrophils) and a variable that described the damage to the epithelium, with the purpose of providing some insight into why different murine strains elicit different immune responses when challenged with the same bacterial load. The authors designated four model parameters to be “strain-dependent” (i.e., varied between mouse strains), and fitted their model to literature-available experimental data corresponding to 4 different mouse strains (CBA/Ca, MF1, BALB/c and C57BL/6) infected with the pneumococcal bacteria. It should be stressed that the four experimental studies used to estimate the parameters of this model, all had different setups and different levels of bacterial load (thus different data available for comparison: all 4 experimental studies had data on lung pathogen levels, while 3 experimental studies had also data on blood pathogen levels and activated phagocytes). Moreover, the authors used some data sets (with lower bacterial load) to obtain a set of parameter values and then validated the models against secondary data sets (with higher bacterial load). Uncertainty analysis was used to show the distribution of both strain-independent and strain-dependent parameters (within defined parameter ranges estimated from the literature), and the principal component analysis was used to identify the most sensitive directions in the parameter space. The principal component analysis results showed that the CBA/Ca mice were most sensitive to the activation rate of neutrophils and to the non-specific clearance rate, the MF1 mice were most sensitive to the non-specific immunity and to the activation rate of neutrophils, BALB/c mice were most sensitive to the blood pathogen phagocytosis rate and to the non-specific immunity, and the C57BL/6 mice were most sensitive to the lung pathogen phagocytosis and the neutrophils activation rate. The numerical simulations also showed higher influx rates of neutrophils for the C57BL/6 and MF1 mice, and lower influx rates for CBA/Ca mice. Since experimental studies suggested that infection resistant mice (BALB/c and C57B/6) have a higher influx of neutrophils compared to mice that do not survive the infection (CBA/Ca and MF1), this results raises a few questions regarding the activation status of the phagocytes, or the potential role of other immune cells (e.g., macrophages) on the outcome of the infection and the survival of mice.
- Malka et al. (2010) derived a 1-equation model for the time-changes in the concentration of some generic bacteria, with nonlinear terms describing growth towards a maximum capacity and death in the presence of neutrophils. The authors created bifurcation diagrams to show that for particular parameter values, the model exhibited either (i) one equilibrium point for the concentration of bacteria (which was inversely proportional to the neutrophil concentration); or (ii) bistability between 2 different levels of bacterial concentration (and for relatively large values of neutrophils). The authors discussed their results in the context of experimental support for bistability phenomena, and in the context of some contradictory results in clinical studies (which might be explained by the existence of this bistability phenomenon). Since the conclusions of the model depended on four assumptions incorporated into the equation for bacterial dynamics (assumptions which might be difficult to test in vivo), the authors also discussed in detail the limitations of their model. The study concluded with the authors emphasising that this simple model, which exhibits bistability behaviour, can be used as a building block in the derivation of other phenomenological, more complex models.

(ii$$^{\prime }$$)
*Models for cell-level dynamics during adaptive response* A large variety of mathematical studies focused on addressing basic questions about T lymphocyte dynamics: from quantifying T cells turnover (Bains et al. 2009; Boer and Perelson 2013) and T cell movement (Beauchemin et al. 2007; Beltman et al. 2009), to quantifying B cell turnover (Hawkins et al. 2007; Callard and Hodgkin 2007), quantifying differentiation patterns of T cells (Gerlach et al. 2013), quantifying asymmetric lineage development in the CD4/CD8 T cell ratio (Sinclair et al. 2013), quantifying cell killing by cytotoxic T lymphocytes (Ganusov and Boer 2008; Gadhamsetty et al. 2015), or maintenance of naive T cell population (Braber et al. 2012; Hapuarachchi et al. 2013). Other mathematical studies investigated analytically and/or numerically the immune response (i.e., T cells and/or B cells) to different bacterial (Ankomah and Levin 2014; Reynolds et al. 2013) and viral infections (Lee et al. 2009; Miao et al. 2010; Huynh and Adler 2012; Luo et al. 2012; Macnamara and Eftimie 2015; Crauste et al. 2015) including autoimmune responses (Blyuss and Nicholson 2012) and immunodeficient responses (Figge 2009), the dynamics between different types of CD4$^{+}$ T cells during allergy (Gross et al. 2011) or during the immune response to cancer (Eftimie et al. 2010b, a; Kronik et al. 2008), or investigate the generation of memory cells and the passive attrition phenomenon (Davis and Adler 2013). Moreover, recent mathematical models have started investigating the differentiation of memory and effector T cells following antigen stimulation (Macnamara and Eftimie 2015; Crauste et al. 2015; Gong et al. 2014). Other models focus on investigating the regulation of the T cell responses (Kim et al. 2007, 2009; Saeki and Iwasa 2010; García-Martínez and León 2010). The majority of these models consider explicit dynamics of the immune response. However, there are also a few models that describe the evolution of different infections and consider the implicit effect of the host adaptive immune response [see, for example, the first model in Luo et al. (2012)].
  We emphasise that the models used to quantify cell kinetics are usually described by simple ODEs, which can be fitted easily to experimental data (thus avoiding the overfitting problems generated by too many parameters). There are also more complex models for cell-level dynamics, which are mainly used for the theoretical investigation of various aspects of the immune response. These models range from classical ODEs [see, for example, Huynh and Adler (2012), Reynolds et al. (2013), Macnamara and Eftimie (2015) and the references therein] and DDEs [to account for the time delay between viral infection and immune response (Lee et al. 2009), or for the time delay between initial CD8$^{+}$ T cell stimulation and full activation (Kim et al. 2007)], to probabilistic models to describe, for example, different probabilities of cell proliferation and death, as in Davis and Adler (2013).
  Next, we discuss in more details to studies: one quantitative study aimed at interpreting labelling data on lymphocyte kinetics, which emphasised the difficulties of interpreting the results and one theoretical study aimed at investigating, only at theoretical level, three hypotheses regarding the factors that affect the dynamics of viral infections that might lead to infectious mononucleosis in young people.

- Choo and Murali-Krishna (2010) combined a simple stochastic model for cell division with murine experiments, to investigate the proliferation and maintenance of memory CD8 T cell population following LCMV infection. With the help of the mathematical model (which calculated the mean number of divisions and variance in the number of divisions of memory CD8 T cells from CFSE data), the authors determined that the proliferation was homogeneous and stochastic, with a small fraction of cells completing division at any given time within an averaged interval of 50 days (this corresponds to a rate of 0.02 divisions/day). Comparison of the memory cells for different epitopes of LMV leads to the conclusion that all memory cells exhibit similar homeostatic turnover characterised by a slow, continuous recruitment into cell division (irrespective of cell specificity and mouse strain) (Choo and Murali-Krishna 2010). Moreover, the authors showed that the homeostatic proliferation of CD8 T cells was independent of CD4 T cell help (the cells being recruited into division in a stochastic manner). The stochastic nature of the turnover in the memory CD8 T cell population was validated by showing that the numbers of divisions follow a Poisson distribution (as predicted by the stochastic model).
  While more and more modes are being derived to quantify various aspects of cell kinetics, the interpretation of the results of these simple models is still a difficult aspect, since the results could depend on the assumptions incorporated into the models, or on the methods/techniques used to obtain them, e.g., the short or long duration of the cell labelling period, deuterium vs. BrdU labelling (Boer and Perelson 2013). The results of these quantitative models have significant consequences on other models for immune responses that use the parameters quantified here. For example, it has been shown that the lifespan of naive T cells differs by a factor of fifty between mice and men, while the lifespan of memory T cells differs by a factor of twenty between mice and men (Boer and Perelson 2013). Therefore, to have any predictive value, the mathematical models that use quantified parameters should make it clear whether they use mice or human data, whether their parameters are fitted to CD4$^{+}$ or CD8$^{+}$ T cells (with the CD4$^{+}$ T cells having a higher turnover), whether the immune data corresponds to acute or chronic immune responses, etc. (Boer and Perelson 2013).
- Huynh and Adler (2012) derived a system of 12 ODE equations for the regulation of Epstein–Barr virus (EBV) infection within a host and the impact on time-dynamics of infectious mononucleosis (IM) in young people. The model was then used to theoretically investigate three hypotheses explaining the high incidence on IM in young adults: increase in saliva and antibodies with age, high cross-reactive T cell responses which vary with age, high initial viral loads (Huynh and Adler 2012). Steady-state and stability analysis (which lead to the calculation of a basic reproductive ratio for viral infection) first identified combinations of parameter values that ensured persistent EBV infections. Then, numerical simulations (which showed the effect of changes in various model parameters on two key measurements of IM: the total number of T cells and the lytic T cell ratio at the peak of the infection) were performed to test the three hypotheses and revealed that the first two hypotheses are supported by the model. The authors concluded that their study highlighted a need for further experimental investigation on the constituents of the saliva that influence infection of B cells and epithelial cells, to help identify thresholds in antibody levels that affect the evolution of the infection.

(iii$$^{\prime }$$)
*Models for Dendritic Cell (DC) dynamics* Dendritic cells are a heterogeneous population of antigen-presenting cells, which receive signals from the environment and, based on these signals, can initiate an appropriate adaptive immune response (by migrating to the draining lymphoid tissues and activating the T cells) (Hugues 2010). Thus, the DCs are a key piece in the innate-to-adaptive immune response. The majority of mathematical models in the literature have been derived to investigate qualitatively and quantitatively various aspects of the interactions between DCs and T cells. The models derived in the past 10 years range from ODE-type equations that focus only on the temporal interactions between DCs and T cells (DePillis et al. 2013; Wares et al. 2015), to delay differential equations (DDEs) that incorporate delays between the DC-T cell contact time and the expansion of T cells (Castillo-Montiel et al. 2015), or agent-based models that consider the spatial structure of the lymph nodes (LN) where the DC-T cell interactions take place (Bogle and Dunbar 2008, 2010). Note that the ODEs can incorporate the spatial aspect of DC trafficking by considering multiple compartments; see the spleen, blood and tumour compartments in DePillis et al. (2013). Other mathematical and computational models described the probabilistic interactions between the antigen-presenting cells and regulatory and effector CD4$^{+}$ T cells inside the LN, in the context of immunity as well as autoimmunity and immunological self-tolerance (Figueroa-Morales et al. 2012; Celli et al. 2012). Some of these models combine computational/mathematical approaches with experimental approaches that involve, for example, flow cytometry and intravital photon imaging, with the purpose of quantifying the number of T cells and DCs to further address questions about their dynamics (e.g., number of DCs required to initiate T cell responses) (Moreau et al. 2016; Celli et al. 2012). Next, we will discuss in more detail the outcomes of such a model.

- Celli et al. (2012) developed a computation model describing the encounters between T cells and DCs in the lymph node (with the T cells performing a 3D Brownian motion, and the DCs being immotile and randomly distributed). With the help of this computational model and an experimental study that measured the efficacy of T cell activation by DCs in vivo, the authors estimated the probability for a T cell to interact with a DC, given a certain number of DCs present in the lymph node. They concluded that fewer than 100 antigen-bearing DCs could be sufficient to initiate a T cell response in a lymph node, when starting from a T cell precursor frequency of 10$^{-6}$. While there are experimental studies which showed a reduced number of DCs in the lymph node that triggered an immune response (Verdijk et al. 2009), there are no experimental studies yet that could test the quantitative prediction made in Celli et al. (2012).
- DePillis et al. (2013) developed a mathematical model (described by 8 ODEs and DDEs) for the interactions between DCs, cytotoxic T lymphocytes and tumour cells. Some of the model parameters were chosen form the published literature, while the remaining parameters were fitted to experimental data from Lee et al. (2007). Then, the authors used this model to explore hypothetical treatment variations: intratumoral DC injections vs. intravenous DC injection; modifying dose timing; the effect of prophylactic vaccines (i.e., vaccine administered prior to tumour challenge). Some of the simulation results (e.g., tumour dynamics following a prophylactic vaccine) showed qualitative agreement with experimental data in. Overall, this approach shows the potential of using modelling to investigate the possible outcomes of various hypothetical scenarios, to gain more understanding on the .

Finally, we emphasise that there are many more mathematical models that investigate the cell-level dynamics of the interactions between the innate and adaptive immune responses following pathogen stimulation, following trauma, or following the injection of cancer cells [see, for example, Vodovotz et al. (2006), Mallet and Pillis (2006), Hancioglu et al. (2007), Marino et al. (2010), Eftimie et al. (2010b), Ankomah and Levin (2014), Cao et al. (2015), Pappalardo et al. (2011) and the references therein]. Other models investigate the interactions between the innate/adaptive immune responses and the pharmacokinetics and pharmacodynamics of specific drugs (Ankomah and Levin 2014). The complex interactions between the innate and adaptive immunity leads to difficulties in parametrising appropriately the models. Next, we will discuss in more detail two models (an experimentally validated model and a theoretical model) that investigate in an integrated manner the innate/adaptive immune responses, to provide some mechanistic understanding of some of the experimentally observed complex immune dynamics.

- Miao et al. (2010) combined modelling approaches with experimental data to quantify the innate and adaptive immune responses to primary influenza A virus infection (for which we lack a detailed and quantitative understanding). To this end, the authors developed a mathematical model to describe the dynamics between the target epithelial cells, influenza virus, cytotoxic T lymphocytes and virus-specific IgG and IgM antibodies. Because of the complexity of the model (described by 15 equations and 48 parameters), many of the parameters could not be measured directly from the data. To address this issue, the authors have split the model into smaller submodels with parameters that can be estimated from experimental data: a model for the initial innate phase and a model for the later adaptive phase. Both models were fitted to experimental data on mice infection with the H3N2 influenza virus A/X31 strain: the first model was used to fit viral titer data between days 0–5, while the second model was used to fit viral titer data between days 5–14, and data on T cell counts and antibody concentrations for the whole period of the experiment. However, before fitting models to the data, the authors performed structural identifiability analyses (Miao et al. 2011) to check whether all parameter values can be uniquely determined from the model and the data and confirmed that this is the case with the exception of one parameter (which, if arbitrarily fixed, will not change the estimated values of all other parameters). For the time period dominated by innate immune response, the authors estimated the half-life of infected epithelial cells to be $\approx$1.2 days and the half-life of free infectious influenza virus to be $\approx$4 h. For the time period dominated by the adaptive immune response, the authors estimated the half-life of infected epithelial cells to be $\approx$0.5 days and the half-life of free infectious virus to be $\approx$1.8 min. The results confirmed that the cytotoxic lymphocytes were crucial in limiting the infected cells, while the antibodies regulated the levels of free virus particles. The authors concluded that this validated model could be further used to predict other aspects of influenza immunity (e.g., the generation of memory CD8$^{+}$ T cells).
- In the context of cancer immunotherapies, Eftimie et al. (2010b) used a mathematical approach to propose a mechanistic explanation behind a surprising experimental observation regarding the anti-tumour effects of CD4$^{+}$ Th2 and Th1 cells: in Mattes et al. (2003) it was experimentally shown that the Th2 cells could reject the B16F10 melanoma in mice, while the Th1 cells could only inhibit tumour growth for a short period of time (which was in contrast to the generally accepted idea that the Th1 cells are the ones eliminating tumours). To this end, the authors developed two ODE models for the interactions between the B16 melanoma cancer cells and the innate and adoptive immune responses described by neutrophils/eosinophils, Th1/Th2 CD4$^{+}$ T cells and cytokines (type-I or type-II, tumour-suppressing and tumour-promoting). The two models (for tumour-Th1-neutrophils interactions via cytokines and tumour-Th2-eosinophils interactions via cytokines) differed in the production rates of cytokines, and in the functions describing the apoptosis of neutrophils and eosinophils, and apoptosis of Th1 and Th2 cells, as controlled by various cytokines. While many of the parameter values were obtained from the published mathematical literature, there were some parameters (e.g., cytokines half lives) which were estimated based on experimental studies and following discussions with immunologists. To clarify the effect that these parameters had on the model outcomes, the authors performed sensitivity analysis (Eftimie et al. 2010b). The results of the models confirmed the experimental observations that the Th2 cells can eliminate the tumour cells in the presence of eosinophils, while the Th1 cells can only reduce for some time the tumour size—but they cannot eliminate the tumour. The suggested biological mechanisms behind this particular tumour-immune outcome was that the rate of tumour killing by eosinophils through degranulation had a more pronounced effect than the rate of tumour killing by tumour-suppressing cytokines (e.g., TNF-$\alpha$, IFN-$\gamma$).

Since many models for cell-level dynamics are described by relatively few equations, it is easier to investigate them using analytical tools (in addition to the numerical simulations). For example, the complex dynamics between some of the components of the adaptive and/or innate immune responses, or between immune cells and tumour cells, has been investigated with the help of stability and bifurcation theory; see for example Webb et al. (2007), Liu et al. (2009) and Foryś (2009). These analytical techniques helped address questions regarding the existence of particular types of states (e.g., periodic solutions that arise via Hopf bifurcations), or questions regarding the possible immunological mechanisms behind the transitions between various states.

## Models for Tissue-Scale Immune Dynamics

In addition to immunological processes that occur inside cells (at molecular level) and between immune cells (at cellular level), there are also immunological processes that occur at tissue level where cells assemble themselves into multicellular structures. Since these tissue-level processes involve interactions between cells, there is sometimes a very fine line between cell-level and tissue-level models (see also Fig. 3). The mathematical models for tissue-level processes are mainly described by PDEs, agent-based or cellular automata models, or hybrid models that combine both PDEs and agent-based approaches—to incorporate the spatial effects of the immune cells on the tissues [see, for example, Su et al. (2009), Sun et al. (2009), Kim and Othmer (2013), Kim and Othmer (2015)]. Nevertheless, there are also a few ODE models that investigate tissue-level processes by ignoring the spatial aspects of these processes and measuring the accumulation of immune cells in the tissues (which can sometimes lead to tissue damage and organ failure, as emphasised by Shi et al. (2015) in a model for immune response to Salmonella infections).

The most common immunological aspects that have been investigated at tissue level are: wound healing (Sun et al. 2009; Cumming et al. 2010; Sun et al. 2009; Adra et al. 2010), tumour-immune dynamics (Su et al. 2009; Kim and Othmer 2015, 2013), the formation of granulomas (Su et al. 2009; Clifone et al. 2013; Fallahi-Sichani et al. 2012), or the formation of micro-abscesses following bacterial infection (Pigozzo et al. 2012). Next we discuss in more detail two mathematical models that emphasise the lack of data (at tissue level) to parametrise models, and the potential use of mathematical techniques (e.g., asymptotic analysis) to gain a deeper understanding of the transitions between different regimes in the dynamics of a biological system.

- As an example of a mathematical model derived to understand a particular aspect of the tissue-level immune response (in the absence of experimental results), we mention the study by Clifone et al. (2013), which used a hybrid model that combined an agent-based approach for the stochastic behaviour of macrophages and T cells in the lung, with ODEs for the dynamics of the cytokines that control the infection (IFN-$\gamma$, TNF-$\alpha$) and those that activate the macrophages (IL-10), and PDEs for the dynamics of chemokines, to investigate the multiscale effects of the cytokines on the formation of granulomas (at the tissue scale) during *M. tuberculosis* infection. The authors first identified a baseline set of parameters which control *M. tuberculosis* infection to levels that were similar to the infection levels observed in various human and non-human primates (some parameters were taken from the published literature, while others were estimated using uncertainty and sensitivity analysis, to match the observed qualitative behaviours). The model was further validated by performing virtual deletion experiments for TNF-$\alpha$, IFN-$\gamma$, and IL-10, and the results—which showed that TNF-$\alpha$ and IFN-$\gamma$ were unable to control disease progression due to a lack of activated macrophages and bactericidal activity, while IL-10 was necessary to control infection—were consistent with previously published experimental data. Then, using sensitivity analysis on the molecular-level parameters related to TNF-$\alpha$ and IL-10 (which, for each cytokine, were grouped in 3 classes: parameters that influenced cytokine synthesis, those that influenced binding and signalling, and those that influenced spatial localisation of cytokines), allowed the authors to confirm that both TNF-$\alpha$ and IL-10 were important in controlling bacterial load and tissue damage. In particular, they showed that a balance between TNF-$\alpha$ and IL-10 was necessary to mediate between the control of *M. tuberculosis* infection and the prevention of host-induced tissue damage, thus defining the granuloma environment. Further computational studies have focused on macrophages polarisation (towards an M1 or M2 phenotype)—as a metric for cytokine signalling—during the progression of *M. tuberculosis* infection (Marino et al. 2015), on the role of IL-10 on lesion sterilisation (Cilfone et al. 2015), or on the designing of various treatments for *M. tuberculosis* infection (Linderman et al. 2015). All these studies were performed only computationally, due to a lack of experimental models of human *M. tuberculosis* infection, and an awareness that the results of the experimental murine and non-primate models existent in the literature might not be reflective of human infections.
- In the context of more theoretical approaches, we discuss next a model for wound healing. While immunity plays an important role in wound healing [with immune cells secreting signalling molecules such as cytokines, chemokines and growth factors, during the inflammatory response (Strbo et al. 2014)], many mathematical models for wound healing treat the immune response in an implicit manner. For example, Flegg et al. (2012) derived a PDE model for wound healing as controlled by oxygen concentration, capillary tip density and blood vessel density. Instead of incorporating explicitly the VEGF dynamics (VEGF=vascular endothelial growth factor—a chemokine important in the inflammation stage of wound healing), the authors assumed that oxygen and VEGF profiles are complementary, and migration up spatial gradients of VEGF would be equivalent to migration down gradients of oxygen. The model equations were first dimensionalised and then simulated numerically for different cases where healing was successful or failed. Next, the authors focused on asymptotic methods to establish conditions under which the growth of new blood vessels can be initiated. These conditions were given in terms of model parameters associated with oxygen supply and oxygen consumption in the wound (and therefore, these conditions depended implicitly on VEGF dynamics—although not included in the model). Bifurcation diagrams were created to show (in a parameter space determined by the oxygen supply and consumption rates), five distinct healing regimes corresponding to successful healing and unsuccessful healing (due to either insufficient and excessive oxygen). The authors concluded their study by comparing the efficacy of different treatments, which were simulated via changes in various model parameters, and the effects of these treatments on shifting model dynamics between different healing regimes. Overall, this theoretical modelling and analysis approach to wound healing lead to a mechanistic characterisation of the transitions between different healing regimes.

We emphasise that many of the models that describe tissue-level dynamics of immune cells are actually multiscale models, since processes that occur in the tissue are the result of molecular and cellular interactions. (We will return to this discussion in the next section.) Due to the complex nature of these models, it is usually very difficult to estimate model parameters, especially since in tissue there are mechanical forces that act among cells and which are never measured and accounted for in these models. The studies that do parametrise these mathematical models generally use parameter estimates done in isolation, via single experiments, or use parameters estimated for different diseases, cell lines and animal models (Flegg et al. 2015). Thus, the results of these models are mostly qualitative.

## Models for Multiscale Immune Dynamics

As mentioned in the previous section, many of the mathematical models that describe tissue-level dynamics of the immune response are multiscale models, since they focus on the role of molecular-level dynamics—such as changes in the components of various signalling pathways, or in the number of cell receptors – on controlling the formation of cellular aggregation structures inside tissues. However, in addition to the models discussed in the previous section, there are many other models that focus on the macro-scale dynamics of the immune cells. For example, in a 2007 review on the multiscale aspect of antigen presentation in immunity, Kirschner et al. (2007) emphasised that while antigen presentation appears to occur only at molecular and cellular scales, the outcome can be affected by events that occur at other scales (e.g., by increased/reduced trafficking of T cells inside the lymph nodes (LN), which might enhance/reduce the opportunity for antigen presentation by DCs). Since multiscale models are being used more frequently to explore the interconnected pathways that control immune responses across different scales (Kidd et al. 2014), in this section we expand the discussion on multiscale models started in Sect. 4, by also including multiscale models that focus on the formation of spatial aggregation structures inside tissues. For a more in-depth review of multiscale modelling in immunology—but with a focus on immunological processes that take place at macroscopic level, which includes both tissue-level models and multicompartment models that describe the movement of cells between organs/tissues/compartments—see Cappuccio et al. (2015).

The majority of multiscale mathematical models in immunology have been developed to investigate phenomena that occur at molecular scale but influence the cell-level dynamics (e.g., cell proliferation, death, cell size, etc.). For example, models have been developed to study the maturation of CD8 T cells in the lymph node as a result of the molecular profile of these cells (as described by TCR and caspase activation, IL-2 production and activation of IL2 receptor, and Tbet protein levels) (Prokopiou et al. 2014); to study the inflammatory response associated with burn injuries (as described by the release of TNF cytokine due to the burn injury, the activation of NF-$\kappa$B pathway, which triggers early, intermediate and late immune responses associated with increased expression of cytokines) (Yang et al. 2011); to study the regulation of NF$\kappa$B signals in the context of macrophage response to *M. tuberculosis* (Fallahi-Sichani et al. 2012); to investigate the movement and activation of immune cells in response to receptor levels and antigen levels (Zheng et al. 2008; Malkin et al. 2015); to study how the balance between IL-10 and TNF-$\alpha$ (and the binding and trafficking of their receptors) influences the formation of granuloma (comprising macrophages and T cells) following *M. tuberculosis* infections (Linderman et al. 2015); or to study the interactions between metabolism (as determined by levels of glucose and insulin produced by $\beta$-cells) and the autoimmune response (caused by macrophages) that lead to the loss of pancreatic $\beta$-cells (Marino et al. 2010).

Another class of multiscale models focused on connecting within-host immunological processes following viral infections to between-host epidemiological models for the spread of the infection throughout a population, thus aiming to understand the effect of population immunity on epidemiological patterns (Feng et al. 2012, 2013; Numfor et al. 2014).

Finally, a completely different class of multiscale models is represented by the kinetic models for active particles (Bellomo and Delitala 2008; Bellomo and Forni 2008; Bianca 2011; Bellouquid et al. 2013; Bianca and Delitala 2011; Kolev et al. 2013; Bellouquid 2014). These models (given by integro-differential equations or partial integro-differential equations) describe the time evolution of heterogeneous populations of cells that have a certain microscopic state (continuous or discrete), which can represent, for example, the degree of activation of a cell, or the degree of cell functionality. In the context of immunology, they have been used mainly to investigate tumour-immune interactions that involve different types of immune cells, as well as mutated (cancer) cells (Bellomo and Forni 2008; Bellouquid et al. 2013; Bianca and Delitala 2011; Bellouquid 2014). However, more recent models have been used to study cytotoxic T lymphocytes (CTL) differentiation (Kolev et al. 2012, 2013) or wound healing (Bianca and Riposo 2015). The complexity of these models makes it difficult to quantify them by fitting the model parameters to the data (since at this moment it is difficult to quantify, for example, the flux/death/proliferation of cells that belong to a subpopulation *i* and have an activity state *j*). Moreover, the complexity of these models does not allow for intensive numerical simulations to investigate large regions of the parameter space. Nevertheless, these kinetic models could be suitable to describe qualitatively the type of experimental data that cannot be quantified at this moment (e.g, data obtained via immunoblotting techniques)—although, to our knowledge, this has not been done yet mainly due to the lack of immunological knowledge of researchers who develop these kinetic models.

Next we discuss in detail two studies of multiscale dynamics for immune responses: one study that combined modelling approaches with experimental approaches to propose a mechanistic framework for the decision of T cells to make extended contacts with DCs and one theoretical study that investigated the link between HIV transmission in a population and the immunity level in a host, and showed how optimal control theory can be used as a tool to reduce the infection at the level of individuals and at the level of population.

- Zheng et al. (2008) used a stochastic model for the spatial aspects of DC-T cell interactions, to understand the behaviour of T cells in the lymphoid tissue in response to the level of pMHC expression. The cells (T cells, DCs that bear pMHC ligands, and DCs that do not bear these pMHC molecules) moved on a lattice representing the lymph nodes, and the movement was described by a Monte–Carlo algorithm. To keep the model relatively simple, the authors did not link their model to an explicit model for signalling pathways, but modelled implicitly the relation between T cells movement/stop responses and antigen concentration with the help of a sigmoidal curve (which described observed TCR-pMHC binding characteristics). With the help of this model, the authors showed that the decision of T cells to stop moving (whose probability was incorporated in the model as part of the Monte–Carlo algorithm) and make stable contacts with DCs, depended on the concentration of pMHC molecules (in a nonlinear manner), on the stability of complexes formed between the cognate peptide and MHC proteins, and on the density of DCs in the lymphoid tissue (in a linear manner). The numerical results of this study were shown to be qualitatively similar with some experimental studies performed in parallel (Henrickson et al. 2008). These combined computational and experimental approaches allowed the authors to propose a mechanistic framework that connected the decision of T cells to make extended contacts with DCs, with the level and type of antigens as well as the ability of T cells to detect the antigen.
- In the context of understanding the effect of population immunity on epidemiological patterns, Numfor et al. (2014) formulated an immuno-epidemiological model that linked a within-host model for the dynamics of HIV particles and infected and non-infected CD4$^{+}$ T cells (described by ODEs), with a between-host model for the dynamics of infected and susceptible individuals in the population (described by ODEs and PDEs). The authors first showed the existence of biologically realistic (i.e., positive and bounded) solutions for this mathematical model. Then, they investigated the local and global stability of the steady states, with the purpose of gaining a better understanding on the long-term behaviour of the system (as controlled by various model parameters). Finally, the authors applied optimal control theory to design intervention strategy for the control of HIV infection based on controlling both the infection transmission rate (between healthy and infected CD4$^{+}$ T cells) and the production rate of HIV virions. The aim was to minimise the number of infectious individuals, the level of free virus particles, and the toxicity of drugs that were given to reduce viral transmission and virion production. A large part of the study was devoted to the rigorous proof of the mathematical machinery that allows for implementation of an optimal control. Numerical simulations have compared the dynamics of the system for two cases: in the presence and absence of drugs that suppress virus transmission and virus production. The drugs lead to an increase in the number of healthy cells, a reduction in the number of infected cells at the host-level and a reduction in the number of infectious cases at the level of the human population.

## Summary and Further Discussion

Mathematical models can provide a valuable framework to organise in a systematic manner immunological concepts, to show the range of outcomes of various immunological hypotheses that cannot be yet tested experimentally, and to generate new mechanistic hypotheses (based on assumptions made regarding the nonlinear interactions among the various components of the complex systems), hypotheses which can then be attempted to be tested experimentally. In this review, we aimed to offer a broad overview of the progress in mathematical immunology over the past 10 years. Due to the extremely large numbers of mathematical models developed during this time, and the large variety of immunological aspects investigated by these models, it was impossible to provide a detailed description of all these models and the subjects covered. Rather, we aimed to emphasise some immunology areas that have been investigated mathematically, the types of mathematical models developed, and the methods used to understand the dynamics of these models. In terms of mathematical models, we remark a shift from simple ODE models to more complex (and sometimes very large) systems of ODEs, stochastic models that require intensive Monte–Carlo simulations, and hybrid and multiscale models that combine ODEs with PDEs and agent-based approaches (Louzoun 2007). However, increased model complexity leads to difficulties in model calibration and model use for quantitative predictions, as well as difficulties to analytically investigate these models. Nevertheless, we need to emphasise that the last 10 years have also seen a shift from a qualitative investigation of immunological processes to a more quantitative investigation of these processes. The development of high-throughput methods to generate new data, as well as the development of immunological methods to quantify available data [e.g., quantification of antigen molecules with flow cytometry (Moskalensky et al. 2015), or detection of antigen-specific T cells (Andersen et al. 2012)] have led to more complex mathematical and computational models that investigate large numbers of interactions (among cells, antigens, cytokines) which occur at different spatial and temporal scales. However, due to the complexity of these new mathematical models they cannot always be fully validated, and the hypotheses generated with their help still have a large qualitative component.

In spite of the very large number of mathematical models developed over the last decade, there are still many immunological aspects not investigated with the help of these models. For example, the recently discovered $\gamma \delta$ T cells [which can be considered a component of both innate and adaptive immunity (Meraviglia et al. 2011)] have not been yet the subject of mathematical modelling and investigation. There are also no mathematical models to investigate the type of innate immune memory associated with macrophages (Yoshida et al. 2015), as well as a few other aspects related to immunological memory [e.g., the role of tissue-resident memory T cells (Mueller and Mackay 2016), regulatory T cell memory (Rosenblum et al. 2016), or the effect of antigen load on memory expansion (Kim et al. 2015)]. There are, of course, many other research directions in immunology where mathematical models could propose hypotheses regarding mechanistic understanding of biological phenomena (and we will mention some of them below, in Sect. 6.3).

For a better understanding of the impact of mathematical models in immunology, in the following we discuss: (1) the benefits of mathematical immunology to date; (2) the opportunities to broaden the applicability of some of the models and analytical methods mentioned in this review; (3) the anticipated trends.

### The Benefits of Mathematical Immunology to Date

Over the last 10 years, various theoretical models have been able to explain existing experimental observations and to generate new hypotheses regarding different immunological phenomena. These theoretical models have ranged from models for T cell receptor signalling and T cell activation (Coombs et al. 2011), to models for T cell and B cell turnover (Boer and Perelson 2013), and models for the immune response during specific infections and their associated therapies (Perelson and Guedj 2015; Canini and Perelson 2014). For example, Canini and Carrat (2011) used a simple ODE model for the kinetics of human influenza A/H1N1 infections and the anti-viral innate immune response mediated by cytokines and NK cells. The model was fitted to individual influenza virus kinetics data obtained from 44 infected volunteers, and the results of the model predicted that the NK cell activity would peak 4.2 days after inoculation (the authors specified that they had no prior data on cytokine or cellular responses, only viral shedding data). Interestingly, an experimental study published in the following year (Pommerenke et al. 2012) confirmed that the NK cell activity during influenza infections peaked around day 5. Since the data were only shown for specific days (e.g., days 3, 5, 8; see Fig. 3 in Pommerenke et al. (2012)) the match between theoretical predictions and data observations seems reasonable. Overall, the majority of mathematical models that have influenced immunology research over the past 10 years were simple models (usually described by ODEs) that could be easily calibrated to experimental data. Nevertheless, also models more difficult to calibrate were beneficial to immunology. For example, the various qualitative models for T cell receptor signalling, such as the kinetic proofreading model that explains pMHC discrimination based on TCR/pMHC bond off-rate (Coombs et al. 2011), have proposed mechanistic hypotheses to shed light on the complex spatial and non-spatial receptor dynamics involved in T cell activation and receptor signalling. Due to a lack of data, these models cannot be confidently parametrised for now (Coombs et al. 2011). Other types of model that have been beneficial to immunology research, despite a lack of model calibration in the absence of relevant data, are the complex systems immunology models that attempt to simulate very large cell signalling pathways (Perley et al. 2014), or models describing complex nonlinear interactions between large numbers of immune cells, cytokines and chemokines (Bianca et al. 2012; Pappalardo et al. 2011; Carbo et al. 2013; Halling-Brown et al. 2010), with the purpose of achieving a global understanding of the possible outcomes of the immune response following small changes in the components (understanding which is difficult to be obtained experimentally due to high costs).

The continuous advances in quantitative experimental techniques (Bandura et al. 2009; Andersen et al. 2012; Newell and Davis 2014), combined with the demand to interpret ever larger and complex data sets to gain a more mechanistic understanding of the immunological phenomena, will eventually lead to new investigative (modelling and analysis) approaches that have better predictive power and will be more readily accepted by the immunological community when designing new studies. On the other hand, the development of new mathematical and computational models (e.g., kinetic multiscale models of active particles) that can use existent data will also help inform the design of new experiments. It is envisaged that mathematical modelling will become more and more intertwined with experimental immunology, in an attempt to answer fundamental questions about how immune system works and evolves over time [thus following the path taken by theoretical ecology, which now relies on sophisticated mathematical- and computer-based models in addition to traditional fieldwork (Otto and Day 2007)].

### Broader Applicability of Some Methods and Models

When thinking about broader applicability of mathematical approaches in immunology, there are two aspects that we need to discuss: (i) broader applicability of certain types of mathematical models and (ii) broader applicability of analytical methods used to investigate specific models.

(i) In regard to the broader applicability of some models, we note that complex multiscale mathematical models (such as kinetic active particle models, or hybrid models) are less likely to be applied widely across different subfields in mathematical immunology. For example, despite the potential of kinetic models for active particles to simulate interactions between cells that have specific traits (e.g., different activation level, different functionality, different markers) (Bellomo and Forni 2008), these models have been generally ignored by the mathematical immunology community since they are more difficult to describe (in particular the integral terms for binary interactions), and more difficult to parametrise. Moreover, as discussed in Sect. 2, the majority of models employed to describe molecular-level dynamics are non-spatial. Nevertheless, advances in experimental techniques have started to reveal the importance of spatial protein dynamics inside cells or on membrane surfaces. It is possible that by focusing only on non-spatial models, one can miss some of the dynamics resulting from spatial movement of proteins, or can incorrectly associate certain non-spatial mechanisms to observed spatial dynamics. As emphasised in Chaplain et al. (2015), simple spatial mathematical models can sometimes exhibit more complex dynamics compared to their non-spatial counterparts and thus can propose different biological mechanisms for the same observed biological pattern [e.g., observed mRNA oscillations in Chaplain et al. (2015)]. It is therefore expected that the next decade will see a wider use of spatial models and multiscale models to gain a better understanding of various immunological phenomena.
(ii) In regard to the broader applicability of some methods, we emphasise that while simple non-spatial ODE models can be investigated using a large variety of analytical methods (ranging from stability and bifurcation theory, to optimisation theory) and can be easily parameterised, more complex agent-based or cellular automata models cannot be investigated as easily due to the lack of specific mathematical methods. Moreover, the knowledge of researchers deriving the mathematical models can impact on the types of analytical methods used to investigate them. For example, while the majority of mathematical models in immunology have been subjected to some sort of local sensitivity analysis (where one parameter is varied at a time within a chosen range), only a few studies focused on global sensitivity analysis using the Latin Hypercube Sampling approach (which allows multiple parameters to be varied simultaneously) (Kirschner et al. 2007). This approach has been applied to both ODEs and agent-based models for immune system dynamics (Kirschner et al. 2007). Another analytical technique that could have broader applicability is the optimal control theory. This technique has been often applied to models in the context of cancer immunotherapies and chemotherapies, to determine the optimal time to administer the anti-cancer treatment, as well as the optimal dose (Castiglione and Piccoli 2006; Ghaffari and Naserifar 2010; Hamdache et al. 2014; Castiglione and Piccoli 2007; Pillis et al. 2007; Pappalardo et al. 2010; Ledzewicz et al. 2012; Itik et al. 2009). However, despite the potential applications to improve therapeutic protocols for various diseases (e.g., diseases caused by viral and bacterial infections), optimal control is not commonly applied to other immunology subfields. Among the few studies that use optimal control theory to improve specific or generic immune therapies, we mention (Tan and Zou 2015) that focused on controlling strategies to enhance the innate immune response to viruses, Stengel (2008) that focused on minimising the HIV viral load and the concentration of infected CD4 T cells, Bayón et al. (2016), Chen et al. (2011) that focused on enhancing various aspects of the innate immune response against some general pathogen, or Numfor et al. (2014) that focused on controlling the transmission rate of HIV infection (between healthy and infected CD4 T cells) and the suppression of HIV virions production with the help of drugs, in an immuno-epidemiological model.
  We emphasise that optimal control approaches combined with experimental approaches could be used successfully to improve current clinical adaptive interventions (Nahum-Shani et al. 2012), with the end goal of designing better personalised patient treatments. Nevertheless, despite the potential of various optimal control approaches to be used in immunology (e.g., to improve the optimal design of clinical trials (Villar et al. 2015), while reducing the costs of these trials), the complexity of mathematical formulation in the context of optimal control makes it difficult for these models to be understood and used by experimentalists and clinicians. Among the few studies that can be applied to clinical trials, we only mention a model describing the probability of not rejecting the null hypothesis, where the optimality of the model (defined in terms of using a minimum sample size) is being investigated using a simple grid search in the parameter space (Mander and Thompson 2010). We believe that more complex optimal control approaches could be used to improve current clinical trials, provided that the researchers involved in these trials are made aware of the bigger picture behind the complex mathematical machinery.

### Anticipated Future Trends

Mathematical immunology is a rapidly evolving field, which continues to follow the development of experimental immunology and at the same time tries to influence it by providing qualitative and quantitative assessments of various immune processes. While the power of modelling and computational approaches in immunology has been recognised in various review studies published in high-impact journals (Chakraborty et al. 2003; Goldstein et al. 2004; Morel et al. 2006; Chakraborty and Das 2010), from an impact point of view the results are still not very encouraging, since these models did not influence significantly the work of experimental immunologists (Andrew et al. 2007).

Despite the fast expansion of mathematical immunology, there are a set of factors that have limited its progress. These factors range from unavailable data to be used by mathematical models, to unavailable models that can interpret existent types of data (e.g., data resulting from Western blots), or more computation power for the numerical simulations of complex models [e.g., 3D agent-based models for particle/cell/protein movement, which sometimes incorporate stochastic rules that require repeated runs to obtain statistical significance (Thorne et al. 2007)]. The progress of mathematical immunology was also limited by an overall lack of interactions between experimentalists and mathematicians. As remarked 10 years ago in Callard and Yates (2005), there is confusion within the general immunology community about how mathematical models can help understand complex nonlinear interactions. Unfortunately, ten years later this confusion still persist (although at a reduced level). On the other side, mathematicians are not always aware of the most recent developments in various immunology subfields that can benefit from modelling, or of the “hidden” questions in immunology that need an immediate answer to be able to move the subfields forward. Neither are they always aware of the amount and type of data that could be available. This lack of awareness might prevent modellers from asking the right questions which, in turn, creates confusion about the value of modelling. Also, when it comes to using data to parametrise mathematical models, mathematicians are often confronted with a multitude of seemingly similar experimental studies which often hold contradictory results. The variety and interpretation of many immunological observations from in vitro and in vivo experiments was also acknowledged by Zinkernagel (2005). Therefore, discussions with experimental immunologists are crucial in this case to decide which data are most appropriate to use for the validation of the model under consideration. In recognition of this necessary approach, recently there have been suggestions to change graduate programmes in immunology to incorporate training in quantitative and computational biology (Spreafico et al. 2015).

It is expected that by removing the limiting factors related to data availability, as well as by tightly integrating the efforts of immunologists and modellers would accelerate the progress in mathematical immunology as well as in experimental and clinical immunology. In particular, this approach will lead to:

(i) the development of new mathematical and computational models (or generalisations of older models) to address the questions considered most important by the experimentalists, thus providing a faster mechanistic understanding of those immunological problems;
(ii) the proposal of new hypotheses regarding the emergent properties of complex immunological systems (Chavali et al. 2008; Krummel 2010);
(iii) the proposal of new, possibly counter-intuitive hypotheses regarding the outcome of nonlinear and non-local interactions between the components of complex immune sub-systems (Chakraborty and Das 2010) [the counter-intuitive aspect of these hypotheses is mainly associated with the limited human brain ability to understand nonlinearity, which determines our focus primarily on linear interactions (Singer 2007)];
(iv) the proposal of mechanistic explanations for some un-intuitive experimental results;
(v) a reduction in (but not complete elimination of) the costs of experiments required to test multiple hypotheses (including a reduction in the use of animals in research—the 3Rs principles for humane experimentation on animals https://www.nc3rs.org.uk/the-3rs), which can then allow a re-allocation of some funds to investigate other research questions;
(vi) the development of mathematical and computational models to help translational immunology research (one of the major limitations of the progress in human immunology being the observed differences between some successful experimental results in mice and poor clinical results in humans (Germain 2010);
(vii) the final use of mathematical and computational models in clinical decision making (e.g., to forecast response to treatment, or to help develop optimal immunotherapy schemes).

The changes we mentioned previously in the context of progress in mathematical immunology will be supported by changes in computational and experimental capabilities. The expected increase in computational power over the next few years will lead to a rapid development of 2D and 3D simulations of immune response in tissues and organs—even for large numbers of components of the immune response (using agent-based, cellular automata, PDE models, or hybrid combinations or these approaches). Comparison between these *in silico* simulations and imaging studies of the immune response [e.g., from lymphocyte activation (Balagopalan et al. 2011), to tracking immune cells in vivo (Ahrens and Bulte 2013), or phenotyping immune cells (Mansfield et al. 2015)] will increase the quantitative understanding of spatio-temporal processes in immunology. The increase in computational power will also allow the incorporation into the models of extremely large numbers of possible complexes that can arise in signalling cascades following the multiple ways proteins can be combined and modified (Goldstein et al. 2004). Finally, possible step changes in the progress of mathematical immunology will likely be associated with the evolution of experimental techniques (Schnell et al. 2012; Köbig et al. 2012; Winter et al. 2015) (e.g., new experimental techniques that could quantify protein levels would lead to a multitude of models for the molecular-level dynamics of these proteins, whose predictions could be tested experimentally).

Since mathematical immunology will continue to follow the developments in immunology, many of the research directions in mathematical immunology that will become most prominent over the next 10 years will follow the main research topics in immunology. A 2011 review article in *Nature Reviews Immunology* (Medzhitov et al. 2011) highlighted some of the future research directions in immunology: understanding the complexities in the development and heterogeneity of macrophages, dendritic cells and T helper cells, as well as understanding the immune processes involved in diseases such as cancer (and their escape mechanisms). Other research directions in immunology that are expected to become prominent in the next years will focus on the development of new vaccines for diseases that do not usually induce robust resistance in infected individuals (Germain 2010) or of vaccines for new infectious diseases, on understanding of metabolic pathways in immune cell activation and quiescence (Pearce and Pearce 2013; Pearce et al. 2013), or on understanding how the immune system is integrated with the endocrine and nervous systems (Kourilsky 2012). Another research direction that will likely become prominent in the next decades is the application of nanotechnology in the field of immunology to improve treatment of various infectious and non-infectious diseases (Smith et al. 2013). The interactions between nanoparticles and various components of the immune system have been shown in some cases to trigger undesirable effects such as immunostimulation or immunosuppression, and more research will be necessary to improve our understanding of these interactions (Zolnik et al. 2010). Also, the upcoming years will see immunology research attempting to integrate the controlled environmental conditions associated with the laboratory experiments, into the variable complex world outside the laboratory (Maizels and Nussey 2013), thus starting addressing questions about the evolutionary processes responsible for observed immunogenic variation, and the importance of the environmental context in various diseases (from parasitic infections, to autoimmunity and cancer). Therefore, from an immunological perspective, it is expected that next decades will see the development of new mathematical and computational models that investigate qualitatively and quantitatively various open questions associated with these prominent research directions in immunology.

From a more mathematical perspective, the research in the next years will likely focus on a few directions, which will include:

- deciding whether to use of stochastic versus deterministic models to better describe certain problems (Heffernan 2011), while taking into consideration the few analytical methods available to investigate stochastic models, compared to the large number of methods available for deterministic models;
- increasing the use of optimal control theory to design optimal treatment strategies in the context of various diseases (Heffernan 2011);
- focusing on the trade-off between complex immunological systems and simple models that can be validated based on existing data (Heffernan 2011);
- increased focus on the development and investigation of multiscale models, to try to understand in an integrated manner the nonlinear interactions between the different components of the immune system which act not only across different spatial scales (from molecular, to cellular, tissue and eventually organ scales) but also across different temporal scales (Cappuccio et al. 2015);
- combining mathematical approaches from evolutionary ecology and immunology to understand the evolution of immunological responses in an environmental-dependent context, thus leading to the further development of the eco-immunology field (Norris and Evans 2000; Garnier and Graham 2014; Serichantalergs et al. 2010);
- in the context of an increase in human infectious diseases outbreaks (Smith et al. 2014), mathematical research will see the further development and investigation of models that link intra-host and inter-host dynamics, with particular applications to the transmission (vector-borne and non-vector-borne) and control of existent and emerging infectious diseases.

To conclude, we emphasise that mathematical immunology is one of the fastest growing subfields of mathematical biology, and the forthcoming years will see this subfield becoming more interlinked with experimental (and eventual clinical) immunology research.

## Appendix 1: Similarities and Differences Between the Main Mathematical Modelling Approaches in Immunology

**Table:**

1.	*Ordinary Differential Equations (ODEs)* are the most mature models. They require a shorter simulation time and can be easily calibrated against available data. For these reasons they are preferred to describe interactions among very large numbers of cells/molecules. ODEs can also be investigated using a variety of mathematical techniques: from uncertainty and sensitivity analysis, to optimal control methods, bifurcation results and existence of bounded solutions (i.e., biologically realistic solutions). Moreover, these types of models have been used to develop various immune simulator platforms, such as COPASI (Hoops et al. 2006), or BioNetGen (Faeder et al. 2009)
2.	*Delay Differential Equations (DDEs)* incorporate various time delays in the reactions. These models are less common in mathematical immunology compared to classical ODEs. The DDEs are also slightly more complex to simulate numerically (compared to the ODEs)
3.	*Stochastic Differential Equations (SDEs)* are derived from ODEs, when the reaction rates between the components of the system are probabilistic. Computationally, these models are slightly more complex than the ODEs. The SDEs are also more difficult to parametrise compared to the ODEs. Also, they are less common in mathematical immunology compared to the classical ODEs
4.	*Partial Differential Equations (PDEs)* are more complex than the ODEs and DDEs and have started to be used more often in the context of modelling spatial and age-related aspects of immune processes. These models require a longer simulation time compared to the ODEs and DDEs and cannot be calibrated very easily (especially when one has to approximate the movement rates of cells/molecules through blood or tissues). Sensitivity analysis can also be applied to PDEs (Kabala and Milly 1991), but this rarely happens for immunology models. Nevertheless, PDEs can be investigated in more detail, due to a large number of analytical techniques available in the literature

**Table:**

5.	*Agent-Based Models (ABMs)* are appealing to non-mathematicians because of the simple, logical rules they incorporate (Chavali et al. 2008). These models can easily account for stochasticity in immunological interactions and individual diversity in a population. However, they are relatively difficult to parametrise. Moreover, these models are computationally very intensive and difficult to analyse mathematically. Sensitivity analysis has been applied to these models (Kirschner et al. 2007), but the approach is still in its infancy. Since ABMs usually try to replicate the complexity of biological systems, they have been used to construct a number of immune simulator platforms, such as NFSim (Sneddon et al. 2011), Kappa (Danos et al. 2007), or ENISI (Wendelsdorf et al. 2011)
6.	*Cellular Automata (CA) models* are appealing to non-mathematicians because of the simple rules they incorporate. The CA models are computationally intensive and more difficult to parametrise and analyse mathematically. Sensitivity analysis could be applied to CA models (Kocabas and Dragicevic 2006), but the approach is still in its infancy
7.	*Hybrid models* are the most complex models, since to simulate them one has to simulate separately their different components (i.e., ODEs, PDEs, ABMs or CA components, using specific methods for each of these components). Therefore, they are computationally intensive. They are also the most difficult to calibrate, since each component has to be calibrated separately using specific methods. Optimisation techniques or sensitivity analysis are almost never applied to these types of models

## Appendix B: Glossary of Mathematical Terms

**Table:**

Glossary	
*Agent-based models (ABMs)* a modelling framework to simulate the dynamics of the components of the system (called “agents”), in discrete time and space. Agents could be individual cells or molecules. This dynamics is described by rules, which are usually literature-derived. The agents are mobile and can interact with other agents or with the environment. Moreover, they can update their states independently of one another	
*BioNetGen* a software for rule-based modelling, which allows for the development of large systems of ODEs, and/or the development of programs that implement discrete-event Monte–Carlo algorithms for simulating stochastic kinetics	
*Cellular Automata (CA)* a modelling framework related to ABMs, which defines a set of rules for updating the state of cells on a grid (this state can be “on” or “off”)	
*Cellular Potts* a lattice-based computational modelling framework that simulates the collective behaviour of (some of) the components of the system (i.e., cells). It uses a set of probabilistic rules to update the interactions among cells (i.e., cell-cell adhesion, cell signalling, cell proliferation, chemotaxis)	
*Complex system* a system formed of numerous interconnected components, which interact with each other. A complex system could exhibit feedback loops and emergent organisation	
*COPASI* a modelling platform used for ODE-based modelling approaches of large systems	
*Deterministic* a system (or a component of a system) whose behaviour is in the future is fully determined by its current state	
*Eco-immunology* an interdisciplinary field that combines aspects of immunology, ecology and evolution, in an attempt to explain natural variation in immune responses	
*ENISI* a modelling platform used for ABM-based modelling of large systems	
*Gillespie algorithm* a type of Monte–Carlo algorithm that generates a realistic trajectory of a stochastic system	
*Hybrid models* models that could combine ODEs, with PDEs, ABMs or CA	
*Immuno-epidemiology* an interdisciplinary field that combines individual (immunological) approaches and population-level (epidemiological) approaches to investigate how differences in individual immune responses affect the population dynamics of parasites or infectious pathogens	
*Integro (partial) differential models* models given by equations that involve both integrals and derivatives of functions	
*Kappa* a modelling platform used for ABM-based modelling approaches of large systems	
*Latin hypercube method (or Latin hypercube sampling; LHS)* a statistical method that generates a sample of parameter values from a multidimensional distribution of values.	
*Monte–Carlo algorithms* are computational methods to simulate the behaviour of a system (or components of a system) for which stochastic fluctuations are important	
*NFSim* a modelling platform used for ABM-based modelling approaches of large systems	
*Ordinary Differential Equations (ODEs)* equations describing the time evolution of some variables representing the averaged concentrations/densities for the components of the system. The models are usually deterministic. They are appropriate to use when the spatial organisation of the components is not important	
*Optimal control theory* an optimisation method that leads to the selection of control policies, based on some defined criteria. It usually reduces to finding the best value of an objective function (best in terms of maximum or minimum), given a set of constraints.	
*Partial Differential Equations (PDEs)* equations describing the time and (usually) space evolution of some variables describing concentrations/densities for the components of the system. They are appropriate to use when the spatial organisation of the components of the system is important	
*Principal component analysis (PCA)* a statistical method that converts a set of possibly correlated variables, into a set of uncorrelated variables called principal components. The first principal component accounts for the most variability in the data, and the second principal component has the second largest variance (being also orthogonal to the preceding component), etc. PCA is usually used as a tool to explore the data	
*Sensitivity analysis* a technique used to quantify the relation between the variation in the input parameters and the effect on model outputs. Could be local (when one parameter is varied at a time) or global (when variations in multiple parameters are investigated simultaneously)	
*Stochastic* a system (or a component of a system) whose behaviour is driven by probabilistic rules. In many stochastic models, the interaction rates between the components of the system are described by probabilistic rules	
*Stochastic Differential Equations (SDEs)* are derived from ODEs that incorporate probabilistic rates for the interactions between system components	
